# Evaluation of Anti-Melanogenesis Activity of Enriched *Pueraria lobata* Stem Extracts and Characterization of Its Phytochemical Components Using HPLC–PDA–ESI–MS/MS

**DOI:** 10.3390/ijms22158105

**Published:** 2021-07-28

**Authors:** Dan Gao, Jin Hyeok Kim, Cheong Taek Kim, Won Seok Jeong, Hyung Min Kim, Jaehoon Sim, Jong Seong Kang

**Affiliations:** 1 College of Pharmacy, Chungnam National University, Daejeon 34134, Korea; gaodan521361@hotmail.com (D.G.); oojh52@naver.com (J.H.K.); kimhm@cnu.ac.kr (H.M.K.); 2 RNS Inc., Daejeon 34014, Korea; happilion@biorns.com (C.T.K.); zmal1329@biorns.com (W.S.J.)

**Keywords:** anti-melanogenesis, stem of Pueraria lobata, molecular docking, B16/F10 melanoma cell

## Abstract

The root of *Pueraria lobata* (Willd.) is a widely used herbal medicine worldwide, whereas the stem of the plant is discarded or used as feed for livestock. To reuse and exploit the stem of *P. lobata* as a resource, we investigated its potential as a skin-whitening agent. We found that the developed, enriched *P. lobata* stem (PLS) extract significantly inhibited melanin production in the 3-isobutyl-1-methylxanthine-induced B16/F10 cells at a concentration of 50 μg/mL. To further confirm the mechanism of the antimelanogenic effect of the enriched PLS extracts, we examined the mRNA expression of tyrosinase, which was suppressed by the extracts. To standardize and implement effective quality control of the enriched PLS extracts, its major chemical constituents were identified by high-performance liquid chromatography–photodiode array–electrospray ionization–mass spectrometry. In total, 12 constituents were identified. In silico analysis showed that the main constituents, puerarin and daidzin, had excellent binding affinities for human tyrosinase. Collectively, our results suggest that the PLS extracts could be used as anti-pigmentation agents.

## 1. Introduction

In mammals, the production and distribution of melanin determine the color of skin, eyes, and hair, which is generated by melanosomes in melanocytes located on the basal layer between the dermis and epidermis [1]. Melanin also plays an important role in protecting the skin from diverse small molecules and stimuli, such as reactive oxygen species, ultraviolet radiation, and cyclic adenosine monophosphate (cAMP)-elevating agents, including forskolin and 3-isobutyl-1-methylxanthine (IBMX) [2]. However, overproduction or aberrant accumulation of melanin may cause many skin hyperpigmentation disorders, including age spots, freckles, and post-inflammatory hyperpigmentation [3,4].

Human skin cells usually produce melanin through a photoprotection reaction. Melanogenesis is a complicated procedure that is regulated through a variety of signaling pathways, and all signals ultimately lead to the upregulation of microphthalmia-associated transcription factor (MITF) expression [5,6]. Melanogenesis begins when activated MITF promotes the tyrosinase gene family expression. Meanwhile, IBMX and other methylxanthines stimulate melanogenesis by inhibiting phosphodiesterase, thereby increasing the levels of cAMP. In turn, cAMP phosphorylates extracellular signal-regulated kinase and phosphoinositide 3-kinase/protein kinase B signaling pathways, ultimately leading to the activation of MITF in the melanogenesis process [2,7,8]. Therefore, evaluating the effects of specific materials on the downregulation of melanogenesis and expression of the tyrosinase gene family could be used to screen potential compounds or extracts used in producing skin-whitening agents.

Currently, kojic acid, hydroquinone, arbutin, and ascorbic acid are known inhibitors of melanin synthesis and have been utilized as skin-whitening agent additives [2,9,10]. However, these compounds have poor penetration into the skin and cause serious side effects, such as skin irritation and skin cancer [11,12]. For these reasons, some countries and regions have restricted the use of these chemicals as additives in cosmetics [13].

*Puerariae Radix*, the root of *Pueraria lobata*, is a widely used herbal medicine in Asia [14]. Pueraria root is also famous for promoting longevity and is used as a royal special food in Japan. In contrast, the *P. lobata* stem (PLS) is mostly used as feed for livestock or discarded, since its medicinal effect is unknown. Previous studies have demonstrated that the root of *P. lobata* is rich in isoflavonoids in the form of glycosides and aglycones, such as puerarin, genistin, daidzin, and daidzein [15,16,17]. The evidence supports the pharmacological and biochemical properties of isoflavonoids, including lowering the risk of cardiovascular diseases, preventing bone mineral loss, and leading to skin whitening [18,19]. As such, the Puerariae Radix extract has been used as a natural source of anti-pigmentation agents used in cosmetic and pharmaceutical industries in South Korea and China [20]. However, harvesting a large amount of the *P. lobata* will destroy the wild ecological environment. Therefore, it is necessary to find new natural resources with whitening effects that can replace *Puerariae Radix*. Previous studies have shown that the roots and stems of *P. lobata* have similar phytochemical composition and that the contents of daidzein and daidzin in the stem are higher than those in the root [21]. The chloroform fraction of the PLS extracts has been shown to have tyrosinase inhibitory effect as it is rich in (-)-tuberosin, isoliquiritigenin, and daidzin [22]. Thus, using PLS as a new material in developing effective whitening cosmetics is a promising approach in cosmetic industry innovation and may also provide a new solution for the development and utilization of discarded PLS resources. In the present study, we aimed to investigate the skin-whitening effect of the enriched PLS extracts and to evaluate their effects on IBMX-induced B16/F10 melanoma cells.

## 2. Results

### 2.1. Effects of the Enriched PLS Extracts on the Viability and Melanin Content of the B16/F10 Melanoma Cells

**Figure 1:**
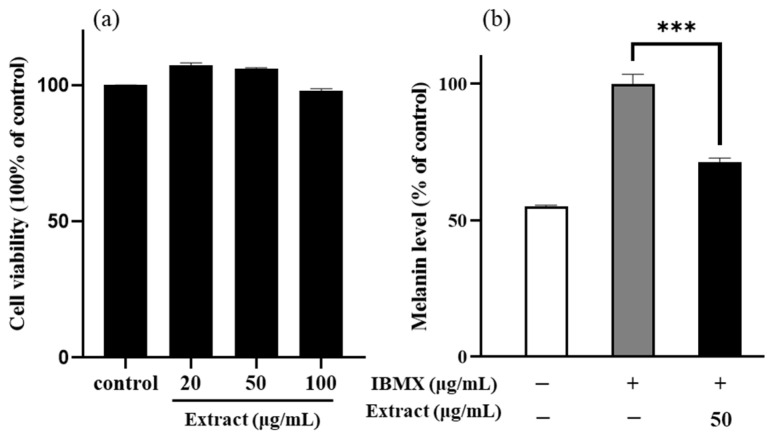
Cell viability (**a**) and melanin content (**b**) in B16/F10 cells treated with enriched PLS extract. *** *p*-value < 0.001 were compared with control group and IBMX induced group, respectively.

To determine whether the enriched PLS extracts has cytotoxic effects, the B16/F10 cells were treated with different concentrations (20, 50, and 100 μg/mL) of the extract for 48 h and cell viability was assessed. The cell viability of the PLS extracts at the tested concentrations was above 97%, indicating no toxicity to the B16/F10 cells, while, at a concentration of 50 μg/mL, the PLS extracts significantly increased cell viability (*p*-value < 0.01, Figure 1a). Therefore, this concentration (50 μg/mL) was used in subsequent experiments.

Next, the B16/F10 cells were cotreated with IBMX and the enriched PLS extract for 48 h, and then the content of melanin was determined to confirm whether the noncytotoxic enriched PLS extracts have an inhibitory effect on melanogenesis. At a concentration of 50 μg/mL, the enriched PLS extracts significantly reduced the melanin content to 71.2% in the IBMX-stimulated B16/F10 cells, relative to the control group (Figure 1b).

### 2.2. Visual Observation of Melanin Pigmentations

To more intuitively reflect the effect of the PLS extracts on the synthesis of melanin in the B16/F10 cells, we performed a visual evaluation to indicate color change in cells treated with the enriched PLS extracts. When compared with the control cells, the IBMX-stimulated cells were dark black, suggesting that IBMX successfully induced melanin production. In addition, the color faded in the cells treated with IBMX and the enriched PLS extracts, indicating that the enriched PLS extracts had a significant whitening effect.

### 2.3. Effects of the Enriched PLS Extracts on the Tyrosinase Expression Levels

**Figure 2:**
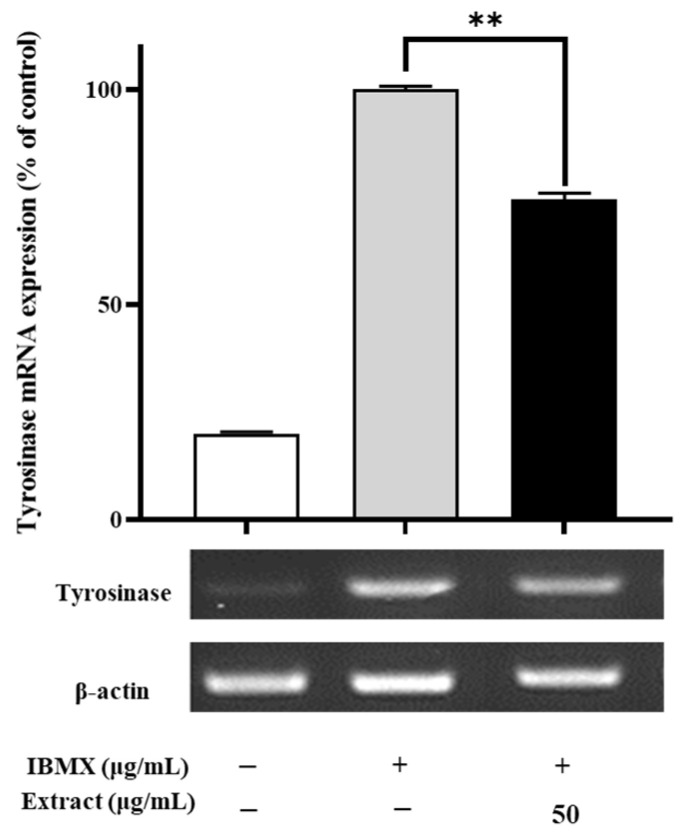
Anti-melanogenesis effect of enriched PLS extracts on mRNA expression. Representative images of PCR products are displayed. The β-actin was used as an internal control for equal loading. Data represent the mean ± SD of three independent experiments. ** *p*-value < 0.01 compared with IBMX induced group.

To examine the possible molecular mechanisms underlying the negative regulatory effects of the enriched PLS extracts, the expression levels of a melanogenesis-related gene (tyrosinase) in the B16/F10 cells were evaluated using real-time quantitative polymerase chain reaction (RT-PCR). As shown in Figure 2, the enriched PLS extracts (50 μg/mL) significantly reduced the mRNA expression of tyrosinase in the B16/F10 cells. After combined treatment with IBMX and the enriched PLS extracts (50 μg/mL) for 48 h, the tyrosinase gene expression levels were significantly reduced to 74.4% compared with the control cells (*p*-value < 0.01), suggesting that the enriched PLS extracts could diminish the biosynthesis of melanin in the B16/F10 cells by inhibiting the expression of tyrosinase. These results indicate that the enriched PLS extracts downregulates tyrosinase at the transcriptional level, suggesting its important role in skin whitening.

### 2.4. High-Performance Liquid Chromatography–Photodiode Array–Electrospray Ionization–Mass Spectrometry (HPLC–PDA–ESI–MS/MS) Analysis

**Figure 3:**
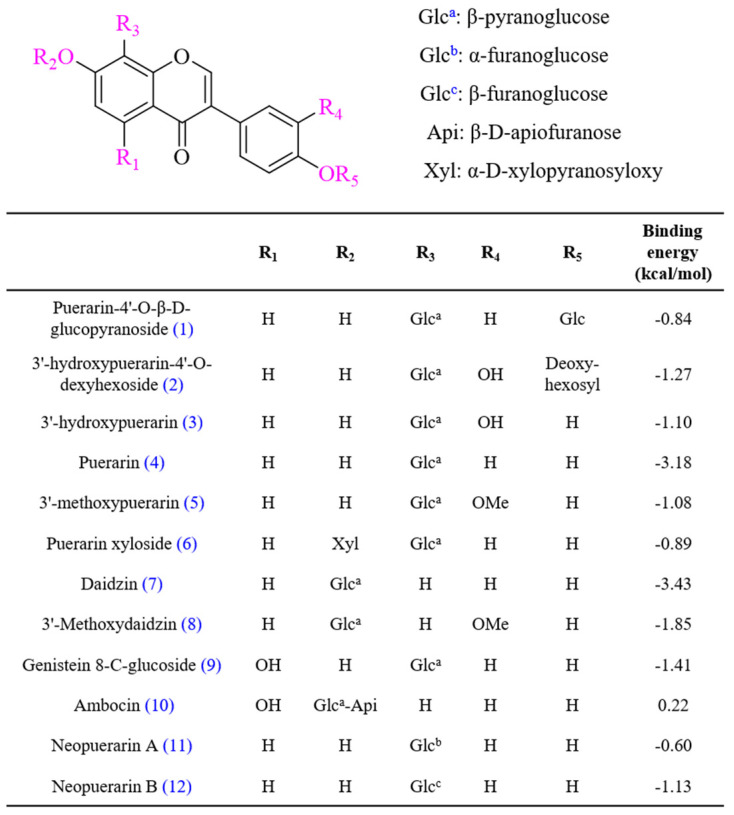
Chemical structures and binding energies of isoflavonoids identified from enriched PLS extract.

To profile the main PLS extracts constituents, the optimized HPLC–PDA–ESI–MS/MS method was applied. The chemical structure of each constituent was identified and confirmed by following procedures: first, the UV spectra of the peaks were obtained using HPLC–PDA in scan mode (200–400 nm), which could be used for discriminating the structure type. Second, the precursor ions ([M+H]^+^ and [M–H]^–^) provided the accurate molecular weights and authentic molecular formulas of these compounds. Third, the MS/MS fragmentation ions provided detailed structure information of the compounds. Finally, 12 compounds were tentatively identified on the basis of their elution order, accurate molecular weight, and fragmentation data or by comparison with previous literature of components isolated and identified in the flowers, leaves, stems, and leaves of *P. lobata* (chemical structures shown in Figure 3).

**Figure 4:**
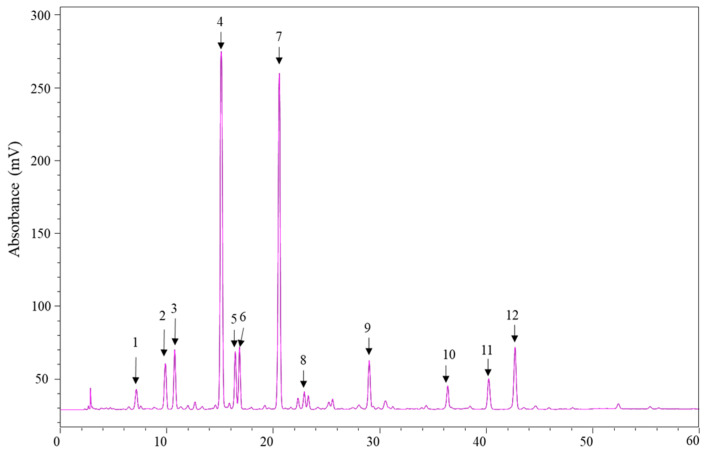
HPLC–PDA chromatogram of identified compounds in enriched PLS extracts monitored at 254 nm.

Figure 4 displays the HPLC–PDA chromatogram detected at 254 nm profiles of the enriched PLS extracts. Peak 4 was confirmed to be puerarin by comparing its maximum UV wavelength, MS spectra, and retention time with that of the reference standard. The UV spectra of Peaks 1–12 were compared with the previously reported results with other parts of P. Lobata (Wild.). All peaks had maximum absorbance between 250 and 300 nm and matched with spectra of isoflavonoids. Thus, based on the ESI-MS/MS and reported literature [22,23,24,25], Peak 1 was determined to be puerarin-4′-O-β-D-glucopyranoside; Peak 2, 3′-hydroxypuerarin-4′-O-dexyhexoside; Peak 3, 3′-hydroxypuerarin; Peak 5, 3′-methoxy puerarin; Peak 6, puerarin xyloside; Peak 7, daidzin; Peak 8, 3′-methoxy daidzin; Peak 9, genistein 8-C-glucoside; Peak 10, ambocin; Peak 11, neopuerarin A; Peak 12, neopuerarin B. Puerarin (Peak 4) and daidzin (Peak 7) were identified as the major components. The retention time, λ max, precursor ions, and product ions are summarized in Table 1.

### 2.5. In Silico Docking

**Figure 5:**
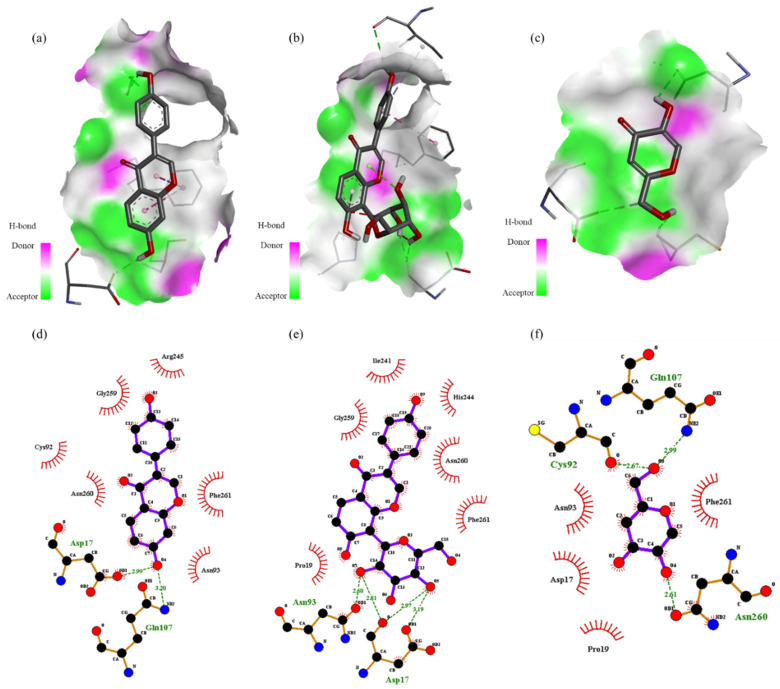
Hydrogen-bonding pocket view of the binding location of daidzin (**a**), puerarin (**b**), and kojic acid (**c**) and predicted binding positions between human tyrosinase (PDB ID:1BUG) and daidzin (**d**), puerarin (**e**), and kojic acid (**f**).

Our in vitro experiments demonstrated the whitening effect of the enriched PLS extracts; however, the constituents contributing to the skin-whitening effect of this extract remain unknown. Therefore, we performed in silico molecular docking to predict the binding affinities and interaction between the phytochemical constituents and the target proteins. The docking affinity scores of the 12 compounds found in the enriched PLS extracts to human tyrosinase are illustrated in Figure 3. Puerarin and daidzin showed strong docking abilities, which are consistent with the results of previous reports [26]. Discovery studio was used to decipher the hydrogen bond pocket view of the interactions between the active compounds (puerarin, daidzin, and kojic acid (positive control)) and human tyrosinase (Figure 5a–c). As shown in Figure 5d, daidzin formed two hydrogen bonds, one between its hydroxyl group and Asp17 at a distance of 2.99 Å and one between its hydroxyl group and Gln 107 at a distance of 3.20 Å. Interestingly, kojic acid, used as a positive control, also formed a hydrogen bond with Gln107 at a distance of 2.99 Å. As shown in Figure 5e and Table S1, puerarin formed four hydrogen bonds between its glucopyranosyl group and Asn93 (at a 2.60-Å distance) and between its glucopyranosyl group and Asp17 (at distances of 2.81, 2.97, and 3.19 Å). Van der Waals interactions were also observed between these active ingredients and human tyrosinase residues (Table S1). For example, we noted Van der Waals interactions between puerarin and active-site amino acid residues Ile241, His244, Asn260, Phe261, Pro19, and Gly259.

## 3. Discussion

The protein kinase A (PKA) signaling pathway plays important roles in melanogenesis and can be activated by IBMX stimulation, which in turn activates adenyl cyclase to elevate cAMP levels [27]. Many transcription factors, such as MITF and cAMP-responsive element-binding protein, can be regulated by the PKA signaling pathway, which plays a key role in the expression of melanogenesis regulators, such as tyrosinase [28,29]. Thus, the IBMX treatment leads to increased expression of the tyrosinase gene family and MITF by the cAMP activation. A previous study found that the Papenfusiella kuromo ethanol extract (40 μg/mL) downregulates melanin in the IBMX-stimulated B16/F10 cells to 65.17%, which was similar to the positive control (kojic acid, 72.30%) [2]. In the present study, the enriched PLS extracts exerted an impressive inhibitory effect on melanogenesis similar to that of the reported herb extract (Figure 1). To assess the skin-whitening effect of the PLS extracts on a gene level, RT-PCR technology was applied to evaluate the mRNA expression of tyrosinase in the B16/F10 melanoma cells.

Melanogenesis is a complex process of melanin production in melanocytes, which involves several enzymatic reactions that convert tyrosine to melanin [5]. Tyrosinase was reportedly involved in regulating melanin formation, and its levels are closely associated with skin pigmentation levels [30]. The expression levels of tyrosinase are modulated by MITF binding to tyrosinase gene promoter, which increases the level of human tyrosinase-related protein-1 and human tyrosinase-related protein-2, in turn increasing the production of melanin. Therefore, inhibition of tyrosinase is an effective way to achieve skin whitening. A previous study showed that a Chlamydomonas reinhardtii extract could downregulate the expression levels of tyrosinase, revealing its potential as a natural whitening resource [31]. In this study, we found that the tyrosinase mRNA expression levels were significantly repressed by the enriched PLS extracts. Our data indicate that the enriched PLS extracts could be used as a novel agent with skin-whitening potential.

Based on previous literature and the use of authenticated standards, we identified the phytochemical constituents of the enriched PLS extracts. Among them, puerarin was the most abundant compound that exerted a good tyrosinase inhibitory effect with an IC_50_ value of 478.5 μM [26]. Puerarin has long been recognized for its beneficial effects on cardiovascular, hyperglycemic, and neurological disorders. Daidzin was found as the second abundant constituent in the enriched PLS extracts that inhibited mushroom tyrosinase with an IC_50_ value of 17.50 μM [22]. Interestingly, our in silico interaction studies showed that puerarin and daidzin have high binding affinities to human tyrosinase. Notably, in the docking experiment, we chose human tyrosinase as the receptor protein instead of mushroom tyrosinase, because only 23% of the amino acid sequence of mushroom tyrosinase is the same as that of human tyrosinase [32,33]. Importantly, the enriched PLS extracts contains several isoflavonoid compounds. Thus, its skin-whitening effect might be due to the concerted action of several isoflavonoid compounds rather than of a single ingredient.

## 4. Materials and Methods

### 4.1. Plant Materials and Reagents

The authenticated stems of *P. lobata* (Willd.) were purchased from Hanpoong Pharm. Co Ltd. (Seoul Korea). Puerarin (99.5%) was obtained from Alfa Aesar (Alfa Aesar, Ward Hill, MA, USA) and used as a reference standard. HPLC-grade acetonitrile and water were obtained from Burdick & Jackson (Muskegon, MI, USA). MS-grade formic acid and IBMX were purchased from Sigma-Aldrich (St. Louis, MO, USA). Dulbecco’s Modified Eagle’s Medium (DMEM), fetal bovine serum (FBS), and penicillin–streptomycin (Gibco BRL, Amarillo, TX, USA) were purchased from BIO-RAD (Sacramento, CA, USA). The EZ-Cytox kit was purchased from Daeil Lab (Chungcheongbuk-do, Korea).

### 4.2. Preparation of Enriched PLS Extracts

*P. lobata* (80 kg) was extracted with 10 times the amount of 30% ethanol at 90–100 °C for 3 h, and the extract solution was filtered through a 5 μm filter. The filtrate was concentrated and dried to obtain a powdered extract. Then, 10 times of distilled water was added to the extract powder, and the same volume of hexane was added to remove fat-soluble impurities, such as pigments. Afterward, ethyl acetate was added twice to the extract, and the two extracts were combined and concentrated at 60 °C to obtain the extract powder (1.53 kg). The extract powder (100 g) was diluted in 7 times the volume of n-hexane and stirred at room temperature for 3 h, after which the mixture was concentrated and dried to obtain the enriched PLS extracts powder.

### 4.3. Cell Culture

The B16/F10 murine melanoma cells were obtained from the American type culture collection (Rock-vile, MD, USA) and cultured in DMEM supplemented with 10% FBS, nonessential amino acids (1%, *v*/*v*), and antibiotics solution (100 U/mL of penicillin and 100 μg/mL of streptomycin) at 37 °C in 5% CO_2_. Stock solutions were prepared at a concentration of 5 mg/mL in dimethyl sulfoxide and were then diluted to appropriate concentrations in the growth medium.

### 4.4. Cell Viability Assay

Cell viability was analyzed using the EZ-Cytox kit assay. In brief, the B16/F10 cells were seeded into a 96-well plate at a density of 5 × 10^4^ cells/well and cultured at 37 °C for 24 h. Next, the culture medium was replaced with fresh medium containing the enriched PLS extract at different concentrations (ranging from 20 to 100 μg/mL) and cells were incubated for 48 h. Subsequently, 10 μL of the EZ-Cytox solution was added into each well and cells were further incubated for 30 min. The absorbance measurement of cells in the absence of any treatment was regarded as 100% cell survival. Each treatment was performed in triplicate and each experiment was repeated three times.

### 4.5. Determination of the Cellular Melanin Contents

The melanin content was determined using a modified method, as previously published [34]. Briefly, the B16/F10 cells (3 × 10^4^) were seeded into a 24-well plate for 24 h and then the DMEM medium was changed with fresh medium containing the enriched PLS extracts at a concentration of 50 μg/mL. After 1 h of incubation, the cells were treated with 100 μM IBMX and further incubated at 37 °C in 5% CO_2_ for 48 h. At the end of the treatment, cells were washed twice with phosphate buffer saline (PBS), dissolved in 400 μL of 1 N NaOH solution at 60 °C for 1 h, and the absorbance was determined at 405 nm using a microplate spectrophotometer (TECAN, Salzburg, Austria). The amount of melanin was expressed as a percentage compared to the control group.

### 4.6. Visual Evaluation of Melanin Pigmentation

The B16/F10 cells were dispensed into a 24-well plate at 3 × 10^4^ cells/well and cultured for 24 h. Then, the culture medium was replaced with fresh medium containing different concentrations of the enriched PLS extracts and incubated at 37 °C in 5% CO_2_ for 48 h. Next, the cells were treated with 100 μM IBMX and further incubated for 48 h. After cultivation, the cells were washed with PBS and centrifuged at 1200 rpm for 5 min to carry out the visual evaluation.

### 4.7. RT-PCR Analysis

Total RNA was isolated from the B16/F10 cells based on the manufacturer’s instructions (SV Total RNA Isolation System, Promega, Medison, WI, USA). The isolated RNAs were reverse transcribed into cDNAs using the First Standard cDNA Synthesis Kit (MBI Fermentas, Vilnius, Lithuania). Reverse transcription and polymerase chain reaction amplification were conducted using an Access RT-PCR System. The synthesized cDNAs were subjected to real-time quantitative PCR, and quantification of the PCR products was performed using the 7500 Real-time PCR System (Applied Biosystems, Foster City, CA, USA) with the SYBR Premix Ex Taq II kit (Takara Bio). The primer sequences designed and used in this study are shown in Table S2. The PCR conditions used are displayed in Table S1. The obtained PCR products were electrophoresed on a 1.5% agarose gel at 100 V for 30 min, and the results were confirmed through UV imaging using Fusion Quick Guide.

### 4.8. HPLC–PDA–ESI–MS/MS Analysis of PLS Extracts

The main components in the enriched PLS extracts were analyzed using HPLC–PDA–ESI–MS/MS (Shimadzu, Kyoto, Japan). Chromatographic separation was carried out using an Optimapark C18 column (4.6 mm × 250 mm, 5 μm) at a flow rate of 1 mL/min. The mobile phase comprising 0.1% formic acid in aqueous solution (Solvent A) and acetonitrile (Solvent B) at a linear-gradient elution program from 10% to 40% of Solvent B for 60 min. For mass spectrometric determination, the HPLC-PDA system was coupled to an LCMS 8040 system equipped with an electrospray ionization interface (Shimadzu, Kyoto, Japan). The wavelength for PDA detection was monitored between 200 and 400 nm. MS was performed in negative and positive modes with an interface voltage set at −3.5 and 3.5 kV, respectively. Other analytical conditions were as follows: drying gas flow, 15 L/min; nebulizing gas flow rate, 3 L/min; desolvation line temperature, 250 °C; heat block temperature, 400 °C. MS^2^ data were obtained using a product ion survey scan in positive and negative modes.

### 4.9. Molecular Docking Simulation

Molecular docking analysis was used to predict the binding affinity of the components found in the enriched PLS extracts to the active site of human tyrosinase (PDB ID: 1BUG; https://www.rcsb.org/structure/1BUG, accessed on 8 July 2021) [35,36]. All chemical structures used as ligands were prepared using ChemDraw 18.0 (CambridgeSoft, Cambridgeshire, UK). Chem3D 18 was applied for the conversion of the 2D structure to 3D, and energy minimization was applied using a force field MM2 approach. The energy to minimize the RMS gradient (0.100) was fit in each iteration. Then, we docked these molecules to the human tyrosinase active site using the AutoDock software package, version 4.2, which is an effective tool for predicting the interaction of prepared ligands with their cognate macromolecular targets and find the global minimum in the interaction energy between the ligand and target protein. All water molecules were deleted, missing hydrogens were added, and non-polar hydrogens were assigned to their corresponding carbons using the AutoDock tools after determining the Kollman united atom charges. The grid box size was set at 60 Å × 60 Å × 60 Å with a grid-point spacing of 0.375 Å, and the center of the grid box was 23.129, 101.558, and 2.507 in the X, Y, and Z center for docking the ligand to the target active site of human tyrosinase [37]. Discovery studio and ligplot (Cambridge, UK) were applied for the visualization of the best-docked conformations.

### 4.10. Statistical Analysis

Statistical significance was analyzed with unpaired Student’s *t*-test and one-way analysis of variance (ANOVA) by GraphPad Prism (Version 8.02, GraphPad Software Inc., La Jolla, CA, USA). * *p*-value < 0.05, ** *p*-value < 0.01, and *** *p*-value < 0.001 were considered to demonstrate statistically significant differences.

## 5. Conclusions

We demonstrated that the developed PLS extracts effectively promotes skin whitening by repressing IBMX-induced melanin synthesis and tyrosinase expression in B16/F10 cells. Further, we identified puerarin and daidzin as the major compounds of the extract and demonstrated their excellent binding affinity to tyrosinase. Consequently, the enriched PLS extracts could be potential skin-whitening agents in cosmetics.

## Figures and Tables

**Table 1:** Peak assignments in the HPLC–PDA–ESI–MS/MS analysis of enriched PLS extract.

Peak No.	RT (min)	Identification	Molecular Formular	λ Max (nm)	[M+H]+	[M–H]–	Product ion (m/z)
1	7.83	Puerarin-4’-O-β-D-glucopyranoside	C_27_H_30_O_14_	248,303	579.20	577.15	297.32, 417.20
2	9.89	3’-hydroxypuerarin-4’-O-dexyhexoside	C_27_H_30_O_14_	249,297	579.20	623.15 [M+HCOOH–H]^–^	433.14, 283.16, 313.39
3	10.88	3’-hydroxypuerarin	C_21_H_20_O_10_	250	433.10	431.10	313.40, 283.20
4	15.43	Puerarin	C_21_H_20_O_9_	250,305	417.10	415.09	297.12
5	16.74	3’-methoxy puerarin	C_22_H_22_O_10_	250,306	447.15	445.10	327.08, 297.07
6	17.33	Puerarin xyloside	C_26_H_28_O_13_	250	549.15	547.15	417.12, 297.06
7	20.72	Daidzin	C_21_H_20_O_9_	250,306	417.15	461.10 [M+HCOOH–H]^–^	255.07
8	23.08	3’-Methoxydaidzin	C_22_H_22_O_10_	248,300	447.20	491.15 [M+HCOOH–H]^–^	285.08
9	29.03	Genistein 8-C-glucoside	C_21_H_20_O_10_	260	433.10	431.10	311.34, 283.21
10	36.82	Ambocin	C_26_H_28_O_14_	249,300	565.20	563.15	311.05, 283.06
11	40.28	Neopuerarin A	C_21_H_20_O_9_	250,306	417.15	415.10	295.38, 267.26
12	42.46	Neopuerarin B	C_26_H_28_O_14_	250,306	417.15	415.10	295.38, 267.26

## Supplementary Materials

The following are available online at https://www.mdpi.com/article/10.3390/ijms22158105/s1, Table S1: Binding sites and docking affinity scores of the constituents identified from enriched PLS extract as determined using Autodock 4.2. Table S2: The sequence of primers and PCR conditions used in this study.

## Abbreviations

PLS	Pueraria lobata stem
cAMP	cyclic adenosine monophosphate
IBMX	3-isobutyl-1-methylxanthine
MITF	microphthalmia-associated transcription factor
RT-PCR	real-time quantitative polymerase chain reaction
HPLC–PDA–ESI–MS/MS	high-performance liquid chromatography–photodiode array–electrospray ionization–mass spectrometry
PKA	protein kinase A
DMEM	Dulbecco’s modified eagle’s medium
FBS	fetal bovine serum
ANOVA	analysis of variance
PBS	phosphate buffer saline
IC_50_	Inhibitory concentration 50
