# Supplementary material for: Evaluation of Anti-Melanogenesis Activity of Enriched Pueraria lobata Stem Extracts and Characterization of Its Phytochemical Components Using HPLC–PDA–ESI–MS/MS
Source: Int J Mol Sci. 2021 Jul 28;22(15):8105. doi: 10.3390/ijms22158105 (PMC8348418; doi:10.3390/ijms22158105)
Supplement: Supplementary file 1 [file ijms-22-08105-s001.zip › ijms-1291778-supplementary.pdf]

International Journal of *Molecular Sciences*

MDPI

Supplementary Materials

Evaluation of anti-melanogenesis activity of enriched *Pueraria lobata* stem extracts and characterization of its phytochemical components using HPLC–PDA–ESI–MS/MS

Dan Gao [1,†], Jin Hyeok Kim [1,†], Cheong Taek Kim [2], Won Seok Jeong [2], Hyung Min Kim[1], Jaehoon Sim [1,*] and Jong Seong Kang [2,*]

[1] College of Pharmacy, Chungnam National University, Daejeon, 34134, South Korea; gaodan521361@hotmail.com (D.G.); oojh52@naver.com (J.H.K.); kimhm@cnu.ac.kr (H.M.K.)

[2] RNS Inc., Daejeon 34014, Korea; happilion@biorns.com (C.T.K.); zmal1329@biorns.com (W.S.J.)

* Correspondence: kangjss@cnu.ac.kr (J.S.K.); jsim@cnu.ac.kr (J.H.S.); Tel.: +82-42-821-5928 (J.S.K.); Tel.: +82-42-821-5938 (J.H.S.)

† Both authors contribute equally.

Table S1. Binding sites and docking affinity scores of the constituents identified from enriched PLS extract as determined using Autodock 4.2.

Compound	Binding energy (kcal/mol)	No. of H-bond	H-bond interacting residues	Van der Waals bond interacting residues
Puerarin	-3.18	3	ASP17, ILE 241	ILE 241, ASN 260, ASN 93, ASN 255, ARG 245, GLN 107
Daidzin	-3.43	3	GLY259, ASP 17, CYS 92	ARG 245. HIS 244, ASN 260, GLY 259, ASN 93, LEU 18
Kojic acid	-4.32	3	HIS 240, HIS 244, HIS 109	ASN 260, MET 258, HIS 274, HIS 88, PHE 261, PHE 114, PHE 270, HIS 118

Table S2. The sequence of primers and PCR conditions used in this study.

Primer	F/R	Sequences	Cycle	Annealing (°C)
Tyrosinase	F	ATC GGC CAA CGA TCC CAT TT	35	57
	R	TAG GTG GAT TGG CTT CTG GG		
β-actin	F	GAT GCC CTG AGG CTC TTT TC	35	57
	R	TCA GCA ATG CCT GGG TAC TA		
